# Profiles of elemental concentrations in human: contribution of X-ray fluorescence to discrimination between healthy and diseased tissues and prediction of alterations in tongue carcinoma

**DOI:** 10.1080/07853890.2021.1896905

**Published:** 2021-09-28

**Authors:** Luísa Zagalo, Pedro Oliveira, Maria João Oliveira, Luísa Gonçalves, Carlos Zagalo, José Brito

**Affiliations:** a Centro de Investigação Interdisciplinar Egas Moniz (CiiEM), Egas Moniz Cooperativa de Ensino Superior, Caparica, Portugal; b Instituto de Ciências Biomédicas Abel Salazar, Porto, Portugal

## Abstract

**Introduction:**

It has been shown that the concentrations of some elements, for example K, Ca, Cu, Fe, and Zn, may differ significantly between the healthy area and the tumour area in the same human tissue [1]. Most studies conducted so far are focussed on specific elements which are a priori known to be involved in physiological or pathological processes, and thus risk neglecting the potential role of the excluded elements in those processes [2]. The role of elements considered in isolation has been questioned because it ignores the important interactions amongst the various elements [3]. However, even when concentrations of various elements are obtained in the same study, comparisons between healthy and diseased tissues, or correlations between the various elements, both intrinsically multivariate, are often implemented with univariate methods, which may result in observed effects or the inability to detect such effects [4]. The methodologies in this study, which complement multielement determinations by X-ray fluorescence spectroscopy (XRF) and X-ray diffraction (XRD) in several types of biological samples, with multivariate data analysis methodologies, provide an important contribute to fill existing gaps in current knowledge of the role elements in such metabolic pathways.

**Materials and methods:**

Samples consisted of five matched pairs (10 samples) of normal and tumour human tongue tissue. In the developing work, the XRF and XRD techniques are applied in the determination of the concentration profile of several elements of interest, in samples of healthy tissue and tongue carcinoma, with the objective of developing a classification system based on the profile of elemental concentrations which allows to discriminate between healthy tissue and carcinoma, and thus clarify the role of these elements in the aetiology of the disease.

**Results:**

Potential differences in Ca, Fe and S were observed. Intrasampling tests determined that samples were inhomogeneous which may affect the ability to discriminate between normal and tumour tissues.

**Discussion and conclusions:**

It is highlighted that the limited number of samples prevents any conclusive findings for now nevertheless results provide areas of focus for upcoming study.

## References

[1] Carla A, Fonseca J, Brito JAA, et al. Serum Zn levels in dysphagic patients who underwent endoscopic gastrostomy for long term enteral nutrition. Nutricion Hospitalaria. 2014;29:359. 24528353 10.3305/nh.2014.29.2.7035

[2] Carvalho ML, Brito J, Barreiros MA. Study of trace element concentrations in human tissue by EDXRF spectrometry. X-Ray Spectrom. 1998;27(3):198–204.

[3] Darvish-Molla S, Al-Ebraheem A, Farquharson MJ. The identification and differentiation of secondary colorectal cancer in human liver tissue using X-ray fluorescence, coherent scatter spectroscopy, and multivariate analysis. Appl Spectrosc. 2014;68(1):79–87. 24405957 10.1366/13-07047

[4] Sohrabi M, Gholami A, Azar MH, et al. Trace element and heavy metal levels in colorectal cancer: comparison between cancerous and non-cancerous tissues. Biol Trace Elem Res. 2018;183(1):1–8. 28795369 10.1007/s12011-017-1099-7

